# Neural Network Prediction of Locomotive Engine Parameters Based on the Dung Beetle Optimization Algorithm and Multi-Objective Optimization of Engine Operating Parameters

**DOI:** 10.3390/s25030677

**Published:** 2025-01-23

**Authors:** Aiqi Dong, Lijuan Liu, Chunce Zhao, Ying Guan

**Affiliations:** 1 School of Railway Intelligent Engineering, Dalian Jiaotong University, Dalian 116000, China; dongaiqi2024@outlook.com (A.D.); liulj@djtu.edu.cn (L.L.); 2 Gear Transmissing Division, CRRC Qishuyan Institute Co., Ltd., Changzhou 213003, China; 3 Zhan Tianyou Honors College, Dalian Jiaotong University, Dalian 116028, China

**Keywords:** altitude, diesel engine, neural network prediction, multi-objective optimization

## Abstract

Altitude has a significant impact on the power and emissions of diesel engines. This paper combines neural network prediction models with artificial intelligence-based multi-objective optimization algorithms to analyze the performance of internal combustion engines for plateau dual-source locomotives operating at different altitudes. The study focuses on the altitude range based on the Laji Line and selects decision variables and output objectives that significantly affect diesel engine performance for joint optimization. First, the diesel engine is simulated and modeled using GT-Power to generate the required dataset. Then, a random sampling method is applied to generate a dataset of 400 operating points from the simulation model. The experimental results show that the neural network prediction model optimized by the DBO algorithm achieves correlation coefficients above 95%. Finally, the NSGA-II algorithm is used for multi-objective optimization. The optimization results indicate that the proposed intelligent optimization method significantly improves the performance of the diesel engine under different altitude conditions, confirming the effectiveness and potential of artificial intelligence optimization algorithms in diesel engine optimization.

## 1. Introduction

Known as the ‘Roof of the World’, the Qinghai–Tibet Plateau is located in the western part of China. The railway infrastructure in the Qinghai–Tibet Plateau region is well below the national average [1,2]. Under these conditions, the Qinghai–Tibet Railway, constructed in this area, is the highest-altitude and longest plateau railway in the world [3,4,5]. According to existing studies, for every 1 km increase in altitude, the average atmospheric pressure and temperature decrease by 9.5 kPa and 4 K [6], respectively. Therefore, conducting research on the diesel engines of locomotives operating in the plateau environment is of great significance for improving the performance of plateau locomotives and promoting the development of the plateau region.

The performance issues of diesel engines in high-altitude areas primarily manifest as incomplete combustion, poor starting performance, reduced power output, difficult emission control, poor fuel economy, overheating, and durability problems. Due to the thin air and low oxygen concentration at high altitudes, these issues become particularly pronounced during the engine’s actual operation, necessitating the development of practical and effective technical solutions. As the altitude increases, the engine output power of locomotives significantly decreases [7]. This is due to the reduction in air density, which leads to fewer oxygen molecules in a given volume of air. Since the combustion process in diesel engines primarily depends on the reaction between oxygen in the air and diesel fuel, thinner air results in incomplete combustion and increased emissions [8,9]. As the altitude increases, temperatures also decrease, which leads to the poorer starting performance of diesel engines, significant ignition delay, and inadequate diesel atomization [10,11]. In summary, the conditions of low pressure, low oxygen, and low temperatures impact various aspects of diesel engine operation, including the air–fuel ratio and fuel injection atomization [12].

To address these issues and improve the performance of plateau engines, scholars in related fields have conducted extensive research, such as exploring alternative fuels [13,14,15,16]. Other methods include the optimization of the turbocharger in response to the factors influencing atmospheric pressure at high altitudes [17,18,19], as well as improvements in the number of fuel injector holes, their structure, and fuel injection strategies [20,21,22]. These optimization methods all require varying degrees of modification to the engine’s structure. However, selecting and optimizing the engine’s calibration parameters, while improving engine performance and simultaneously achieving emission reduction goals, is currently the most appropriate approach [23,24]. Kamyar Nikzadfar et al., using evolutionary algorithms and artificial neural network-based modeling calibration methods, effectively reduced fuel consumption, emissions, and both the time and cost of the calibration process for diesel engines across all operating conditions [25]. Anuj Pal et al. proposed a machine learning-based calibration framework that efficiently optimized the performance of turbocharged diesel engines equipped with the eBoost system [26]. P. Prakash et al. verified the feasibility of grape biodiesel as a sustainable and economical alternative to conventional fuels by optimizing key parameters. The optimized conditions achieved low emissions and high-efficiency engine operation [27]. Vinay Prakash Chaudhary et al. optimized the load, fuel blend ratio, and hydrogen substitution ratio using the response surface method, achieving higher thermal efficiency and lower emissions [28].

To meet the emission restrictions for diesel engines, the controllable parameters in modern diesel engines have become increasingly complex. With the continuous development of large-scale artificial intelligence models, such as ChatGPT, AI algorithms are gradually being applied across various fields, creating opportunities for developments in each sector [29]. Therefore, using machine learning and artificial intelligence algorithms to optimize engine power and emissions is an excellent choice. Sheng Gao et al. employed the RSM-NSGA III-TOPSIS method to optimize the performance and emission characteristics of blended fuels in high-altitude environments [30]. Aaron M. Bertram et al. demonstrated significant superiority in optimizing diesel engine performance and reducing emissions using an improved hybrid particle swarm optimization–genetic algorithm [31]. Esmail Khalife et al. were the first to model the performance and emissions of diesel engines using blended emulsion fuels with four different algorithms. The results showed that the Gray Wolf Optimization algorithm performed best in both performance and emission predictions [32]. Yuhua Wang et al. proposed a multimodal fusion model combining the improved Seagull Optimization Algorithm (AMSO), which significantly enhanced the prediction accuracy of diesel particulate filter regeneration conditions and emission performance [33].

In 2023, Jiankai Xue et al. proposed a novel biomimetic optimization algorithm based on the foraging behavior of dung beetles, known as the Dung Beetle Optimizer (DBO) [34]. The algorithm, by simulating the foraging mechanism of dung beetles, demonstrates excellent search capabilities. N. Srinivas et al. were the first to propose a non-dominated sorting genetic algorithm (NSGA) for solving multi-objective optimization problems; however, the algorithm also has certain limitations [35]. To address these shortcomings, Kalyanmoy Deb et al. significantly improved the efficiency and effectiveness of multi-objective optimization by introducing the fast non-dominated sorting method, an elitist preservation strategy, and a parameter-free niche operator [36].

With the increase in economic and industrial activities in high-altitude regions, the performance issues of diesel engines have become a critical technical challenge that needs to be addressed. Although some studies have explored optimization methods for diesel engines, most existing methods focus on low-altitude or standard environments, overlooking the impact of specific conditions at high altitudes, such as low oxygen levels and fluctuating air pressure, on engine performance. The shortcomings of these studies include the following: firstly, existing technologies mainly focus on improvements in either combustion efficiency or emission control, lacking comprehensive solutions; secondly, although some methods have achieved ideal results in laboratory settings, they face significant technical difficulties and high costs in practical applications. The goal of this study is to fill these gaps by proposing a novel, comprehensive optimization approach that addresses the unique challenges of high-altitude environments, performing multi-objective optimization targeting both emissions and power output.

In previous studies, due to the experimental difficulty of simulating plateau conditions, building experimental test rigs under different altitude conditions would require extensive modifications to the existing experimental equipment and conditions, which is highly challenging in practical engineering. Furthermore, directly applying the experimental methods from this study in real-world engineering requires substantial financial investments, significantly increasing the cost of experiments. Considering the reliability of the experimental methods, as well as potential risks related to the long-term durability of the engine and emission control during implementation, using GT-Power was selected as a more feasible solution. It can overcome the limitations of environmental conditions, analyze the diesel engine’s performance under varying altitudes, temperatures, and air pressures, and control different parameters to obtain simulation data for optimization objectives, while also reducing the cost of data acquisition.

This paper addresses the performance and emissions of the internal combustion engines in current plateau dual-source locomotives at different altitudes. At first, simulations were conducted using GT-Power, and output data predictions and multi-objective optimization were performed by combining Matlab with machine learning and artificial intelligence algorithms. The use of a neural network prediction model can improve the efficiency of obtaining output results from the diesel engine simulation model after each parameter adjustment. By applying multi-objective optimization to adjust the key parameters of the diesel engine, the model can overcome the impacts of high-altitude environments, thus optimizing both engine performance and emissions. The DBO algorithm used in this study is applied, for the first time, to the neural network prediction of the diesel engine simulation model. The neural network prediction model optimized by the DBO algorithm is also being used, for the first time, in conjunction with the NSGA-II algorithm for multi-objective optimization.

The paper is organized as follows: Section 2 introduces the methods and research process used in the study. Section 3 discusses the methods for acquiring diesel engine data and the establishment of the prediction model. Section 4 provides a detailed explanation of how the DBO algorithm optimizes the neural network. Section 5 presents the application of the NSGA-II multi-objective optimization algorithm, utilizing the neural network prediction model to optimize the diesel engine. Section 6 summarizes the results of the research and presents the conclusions.

## 2. Optimization Methodology

**Figure 1:**
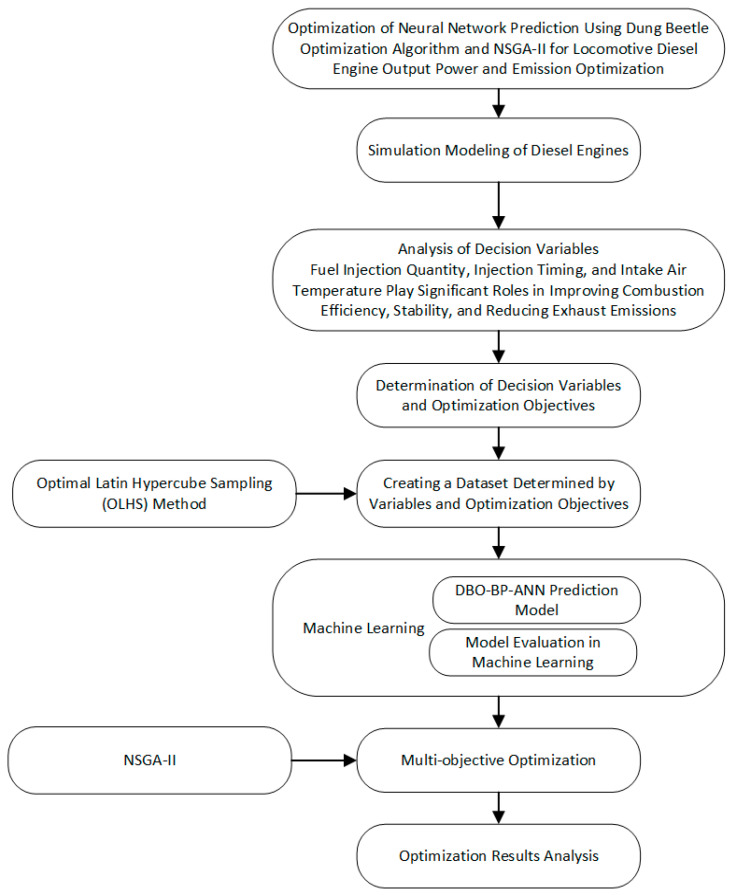
Flowchart of the overall research process.

This study predicts the brake power and emissions of locomotive diesel engines by combining machine learning optimization algorithms with neural networks to model key engine parameters. The NSGA-II algorithm is used for multi-objective optimization. The research process is illustrated in Figure 1. After establishing the diesel engine simulation model and performing model validation, the model is encapsulated for joint simulation with Matlab. The Optimal Latin Hypercube Sampling (OLHS) method is used to obtain the dataset, and an artificial neural network prediction model optimized by the DBO algorithm is developed. The selected decision variables are then used to predict the diesel engine’s output power and emissions. Finally, the optimization of engine brake power and nitrogen oxide emissions is performed by combining the neural network prediction model with the NSGA-II algorithm. The decision variables include INJ (fuel injection quantity), SOI (start of injection), and ICT (intercooler temperature), with the goal of maximizing the diesel engine brake power and minimizing nitrogen oxide emissions.

## 3. Diesel Engine Simulation Modeling and Prediction Optimization Process

### 3.1. Establishment of the Virtual Diesel Engine Model

**Figure 2:**
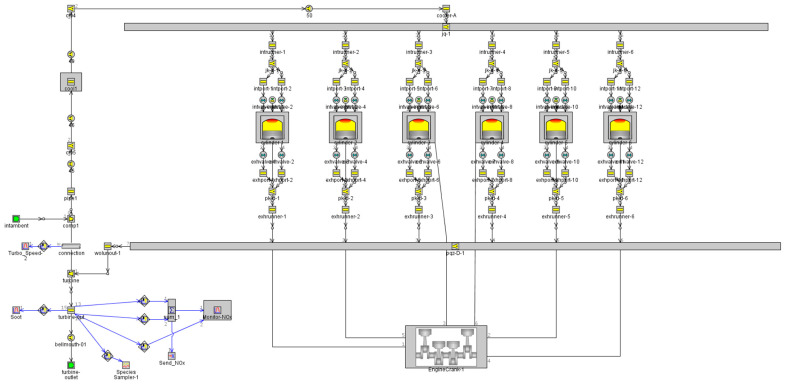
Diesel engine 1D simulation model.

**Figure 3:**
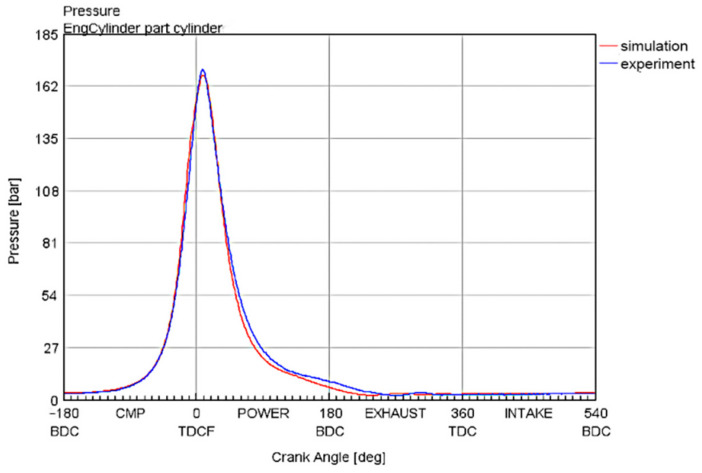
Comparison of pressure curves within the cylinder.

The diesel engine parameters selected in this study are shown in Table 1, with the structural parameters derived from a twelve-cylinder diesel engine used in a high-altitude dual-source locomotive. A one-dimensional simulation model of the six-cylinder diesel engine, as shown in Figure 2, was established using GT-Power. To verify the reliability of the model, the cylinder pressure curve measured by pressure sensors on the test bench for this engine model was compared with the simulation model, as shown in Figure 3. The high degree of correlation between the two curves demonstrates that the model developed using GT-Power is reliable. The data obtained from the internal combustion engine simulation model was coupled with Simulink to create a dataset for the neural network prediction model, which is then used to develop the neural network model for the diesel engine. The input variables for the model include INJ, SOI, and ICT, while the output variables are the NOx mass fraction and engine output power.

### 3.2. Range of Decision Variables and Selection of the Neural Network Prediction Model

According to previous studies, the INJ, SOI, and ICT of the diesel engine have significant effects on improving combustion efficiency and stability, and reducing exhaust emissions. Therefore, these three parameters were selected as the decision variables for the entire optimization process, with the diesel engine’s brake power and NOx mass fraction as the target outputs.

The three parameters selected as decision variables need to have corresponding safety upper and lower limits. Therefore, based on the rated parameters provided by the diesel engine manufacturer, the values of the decision variables are constrained as follows:

(1) $$\begin{array}{rr} & 1200\leq INJ\leq 1800\\ & -16\leq SOI\leq -11\\ & 320\leq ICT\leq 340 \end{array}$$

In addition, the experiment focuses on the internal combustion engine of the plateau dual-source locomotive. The altitudes selected for the operating environment of this engine model are 0 m, 1000 m, 22,000 m, 2800 m, 3500 m and 4000 m, as shown in Table 2.

The backpropagation neural network (BPNN) is capable of modeling complex nonlinear relationships and system behaviors, making it an effective method for addressing the complex dynamic system of a diesel engine. Based on the aforementioned input and output variables, a BPNN is established. The output of the artificial neural network is determined by the network structure, weights, biases, and activation functions, and can be expressed as follows:

(2) $$y=f\left(\sum_{j}w_{ij}x_{j}+b\right)$$

where f is the activation function, w represents the weights, x is the input vector, and b is the bias value.

### 3.3. Acquisition of the Engine Dataset

**Figure 4:**
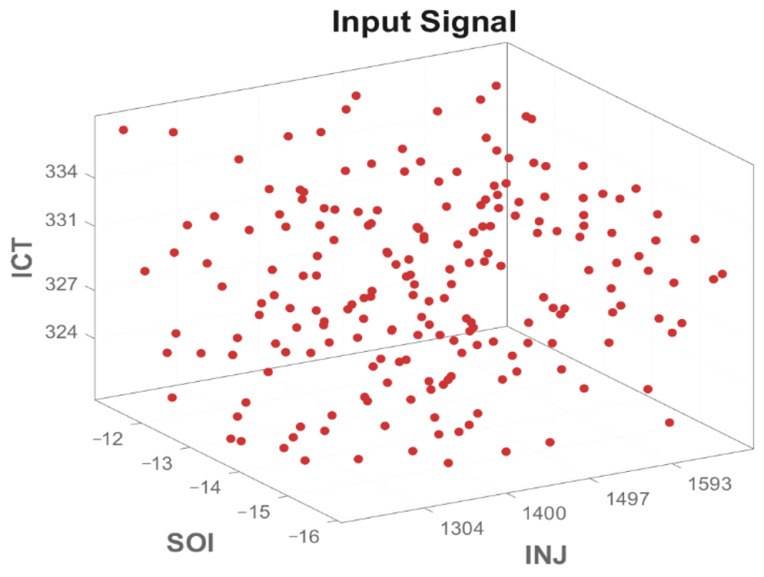
Optimal Latin Hypercube Sampling diagram.

In this study, a virtual engine model is first established using GT-Power2016 software based on the selected engine model parameters and experimental data. The sampling method used to obtain the input samples of the dataset is the Optimal Latin Hypercube Sampling (OLHS) method. This sampling method ensures the uniformity and representativeness of the samples within the defined range of values, particularly in high-dimensional spaces, where it effectively reduces the correlation and concentration between samples. It avoids the local bias caused by simple uniform sampling, thereby making the selected sample points more representative and well covered. Compared to other sampling methods (such as Latin Hypercube Sampling, Monte Carlo Sampling, uniform random sampling, and grid sampling), OLHS offers a more uniform and optimized sample distribution, which provides significant advantages in high-dimensional problems. The dataset generated using this method is shown in Figure 4. The input data include parameters such as INJ, SOI, and ICT. Subsequently, the simulation model is run to obtain the corresponding output dataset, which includes the engine’s brake power and nitrogen oxide (NO_x_) emissions.

Before generating the dataset, in order to ensure the quality of the data samples and provide cleaner and more accurate data for subsequent modeling, data preprocessing is required. Considering that the selected parameter values vary greatly, normalization is necessary. Given that the activation function used for the hidden layer neurons in the neural network is the sigmoid function, Min-Max Scaling is chosen for the normalization and is expressed as follows:

(3) $$\begin{array}{r} X^{\prime }=\frac{X-X_{min}}{X_{max}-X_{min}}\times \left(B-A\right)+A \end{array}$$

where *X* is the original data, *X*_min_ and *X*_max_ are the minimum and maximum values of the data, respectively, *A* and *B* are the upper and lower bounds of the target range, and *X*′ is the normalized data.

Finally, these data are used to construct the training and testing sets for the BP neural network, and based on this dataset, a prediction model for the BP neural network is generated.

### 3.4. Impact of Decision Variables on Target Outputs

**Figure 5:**
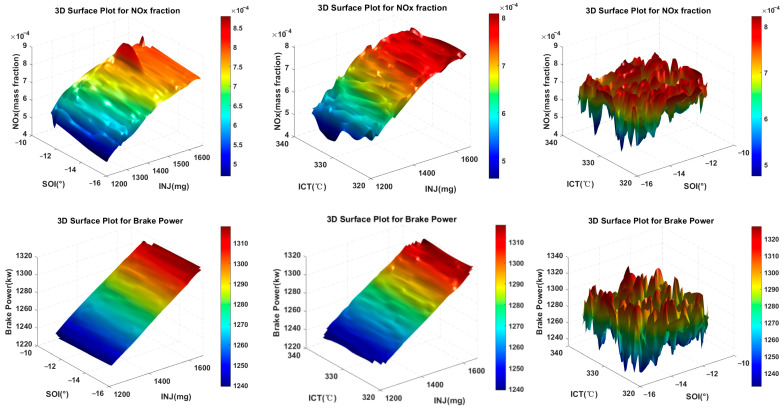
Impact of decision variables on engine performance.

The selection of excitation signals has a significant impact on the predictive performance of the neural network model. To ensure that the neural network can accurately learn and predict engine performance, the input signals should fully represent the key characteristics of the system. As shown in Figure 5, the collaborative effects of INJ, SOI, and ICT on engine performance are examined at an RPM of 1000 r/min and an altitude of 1000 m. In the figure, although no significant linear relationship is observed between the inputs and outputs, some clear trends can still be identified: as INJ increases, NOx emissions initially rise and then decrease, while the brake power is generally positively correlated with INJ. Additionally, SOI and ICT show peak values in their effects on NO_x_ and brake power, with corresponding trends of increase or decrease.

### 3.5. Neural Network Prediction Model Structure

This study adopts the BPNN model as the predictive model for engine performance, and coding and model training are conducted on the Matlab platform. The dataset generated by the GT-Power simulation model is divided into a training set and a testing set, accounting for 70% and 30% of the total dataset, respectively. However, during the training process, solely relying on the BPNN model for prediction may lead to common issues, such as overfitting, excessive training time, and convergence to a local optimum. To prevent the BP neural network from falling into a local optimum, the Optimal Latin Hypercube Sampling method is employed for random sampling, ensuring the diversity and generality of the input data. Additionally, to address other issues in the training process, the distributed DBO algorithm is introduced to optimize the BPNN. This optimization strategy not only effectively increases the training speed but also significantly improves the accuracy of the predictive model. The model is evaluated based on its absolute error, relative error, mean absolute percentage error (MAPE), coefficient of determination (R^2^), mean squared error (MSE) and mean absolute error (MAE). The formulas for these metrics are as follows:

(4) $$Absolute\;Error=y-\hat{y}$$

(5) $$Relative\;Error=\frac{y-\hat{y}}{y}\times 100\%$$

(6) $$MAPE=\frac{\sum_{i=1}^{n}\left|\frac{y_{i}-\hat{y_{i}}}{y_{i}}\right|}{n}\times 100\%$$

(7) $$R^{2}=\frac{{\left(n\sum_{i=1}^{n}{\hat{y}}_{i}y_{i}-\sum_{i=1}^{n}{\hat{y}}_{i}\sum_{i=1}^{n}y_{i}\right)}^{2}}{\left(n\sum_{i=1}^{n}{\hat{y}}_{i}^{2}-{\left(\sum_{i=1}^{n}{\hat{y}}_{i}\right)}^{2}\right)\left(n\sum_{i=1}^{n}y_{i}^{2}-{\left(\sum_{i=1}^{n}y_{i}\right)}^{2}\right)}\times 100\%$$

(8) $$MSE=\frac{1}{n}\sum_{i=1}^{n}{\left({\hat{y}}_{i}-y_{i}\right)}^{2}$$

(9) $$MAE=\frac{1}{n}\sum_{i=1}^{n}\left|y_{i}-{\hat{y}}_{i}\right|$$

where *y* and $\hat{y}$ represent the target values of the training predictive model and the predicted values from the artificial neural network model, respectively, including the brake power and the mass fraction of NOx. *n* represent the number of data points.

Based on the characteristics of the neural network prediction model studied in this paper, the R^2^ and MAE metrics are selected to assess the reliability of the neural network prediction model.

#### 3.5.1. The Prediction Performance of the Traditional BPNN

The BPNN was chosen as the main prediction model. It adjusts the weights and biases in the network through the backpropagation algorithm, which has powerful learning capabilities. It can fit complex linear relationships and, when faced with complex data, automatically adjusts parameters through training to adapt to various intricate models. However, it also has some drawbacks, such as the tendency to get trapped in local optima and its high computational cost. Therefore, the DBO algorithm is integrated to mitigate these issues.

**Figure 6:**
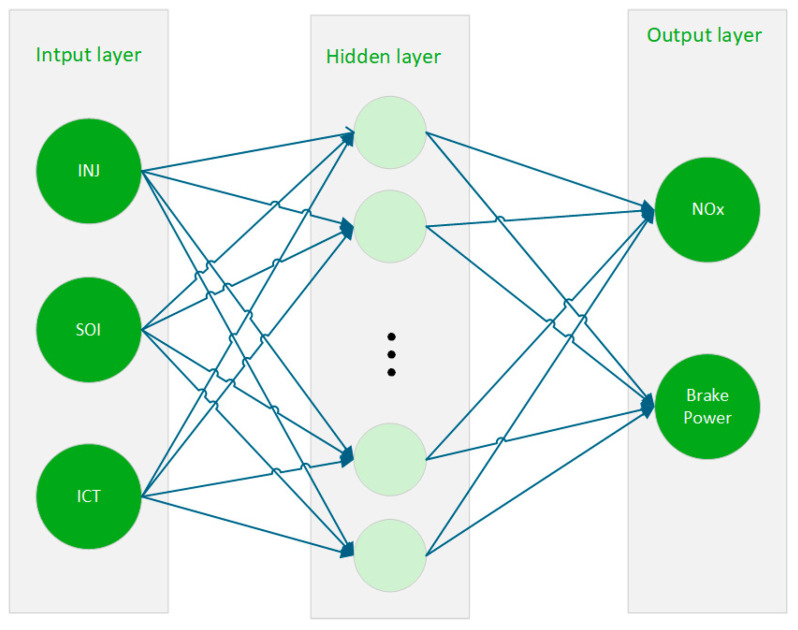
Topology of the BPNN.

The BPNN consists of an input layer, a hidden layer, and an output layer. The topology of the constructed BP neural network is shown in Figure 6.

The specific steps are as follows:

(1) First, initialize the structure of the neural network and determine the input and output dimensions. At the same time, initialize the weights and biases between the input layer, hidden layer, and output layer. Set the number of training iterations, target error, and learning rate. The activation function of the hidden layer is set to sigmoid, and the function expression is as follows:
  (10) $$\sigma (x)=\frac{1}{1+e^{-x}}$$
(2) Transfer the input data of the selected training set to the input layer of the neural network, and compute the outputs from the input layer to the hidden layer and from the hidden layer to the output layer.
  (11) $$z_{1}=W_{1}x+b_{1},\;a_{1}=\sigma \left(z_{1}\right)\;z_{2}=W_{2}a_{1}+b_{2},\hat{y}=\sigma (z_{2})$$
  *W*_1_ and *b*_1_ represent the weights and biases for calculating the hidden layer, *W*_2_ and *b*_2_ represent the weights and biases for calculating the output layer, and $\hat{y}$ represents the predicted results of the model.
(3) Calculate the error between the predicted values and the actual expected values.
  (12) $$\delta_{2}=\frac{\partial L}{\partial \hat{y}}=\hat{y}-y,\delta_{1}=\left(W_{2}^{T}\delta_{2}\right)⊙f\prime \left(z_{1}\right)$$
  *δ*_2_ is the error of the output layer, and *δ*_1_ is the error of the hidden layer.
(4) After calculating the gradients of the weights and biases for the output layer, compute the gradients of the weights and biases for the hidden layer.
  (13) $$\frac{\partial L}{\partial W_{2}}=\delta_{2}\cdot a_{1}^{T},\;\;\;\;\frac{\partial L}{\partial b_{2}}=\delta_{2}\;\frac{\partial L}{\partial W_{1}}=\delta_{1}\cdot x^{T},\;\;\;\;\frac{\partial L}{\partial b_{1}}=\delta_{1}$$
(5) Update the weights and biases in the network.
  (14) $$W_{1,2}\leftarrow W_{1,2}-\eta \cdot \frac{\partial L}{\partial W_{1,2}},\;b_{1,2}\leftarrow b_{1,2}-\eta \cdot \frac{\partial L}{\partial b_{1,2}}$$
(6) Stop the loop when the target value is reached or the maximum number of iterations is satisfied.

**Figure 7:**
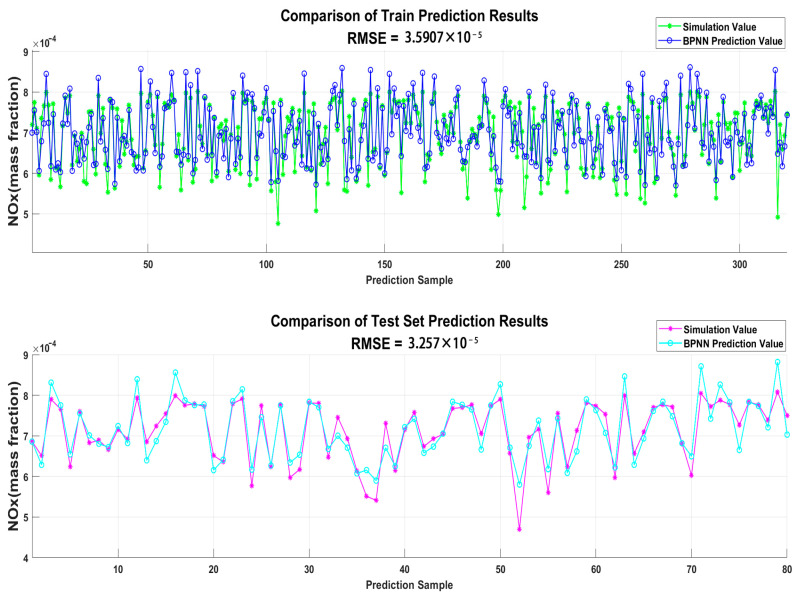
Comparison of BPNN prediction results for NOx.

**Figure 8:**
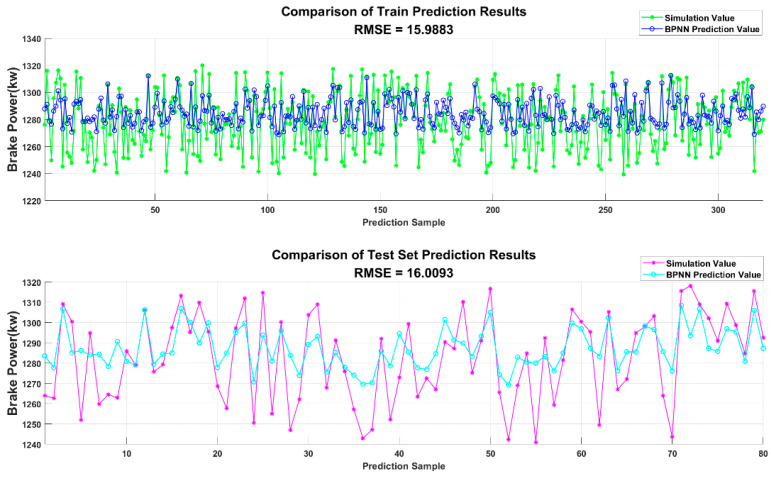
Comparison of BPNN prediction results for brake power.

The BPNN was used to predict the diesel engine dataset samples. The prediction results are shown in Figure 7 and Figure 8. As seen in the figure, the traditional BPNN does not provide ideal prediction results for the diesel engine dataset. The data fitting is relatively poor, and it is insufficient to meet the standards for establishing a prediction model.

#### 3.5.2. DBO Algorithm Combined with BPNN for Neural Network Model Prediction

Since the performance of the traditional BPNN is not satisfactory, we attempt to optimize it using the DBO algorithm. The use of the DBO algorithm can effectively replace the gradient descent method traditionally used in BPNN, especially when dealing with complex and non-convex loss functions. By simulating the foraging behavior of dung beetles, DBO can perform global searches, helping the neural network find better solutions. The DBO algorithm optimizes the weights and biases within the neural network, aiding the model in making more accurate predictions. The detailed optimization process is explained in Section 4.2.

In determining the number of hidden layer neurons, a simple trial-and-error optimization method is used due to the small range and low cost of trial-and-error. Table 3 shows the average training time and R^2^ for different numbers of hidden layer neurons, using the brake power neural network training model as an example. From this, it can be concluded that the number of hidden layer neurons should be set to 10, as it yields the highest efficiency and fit.

**Figure 9:**
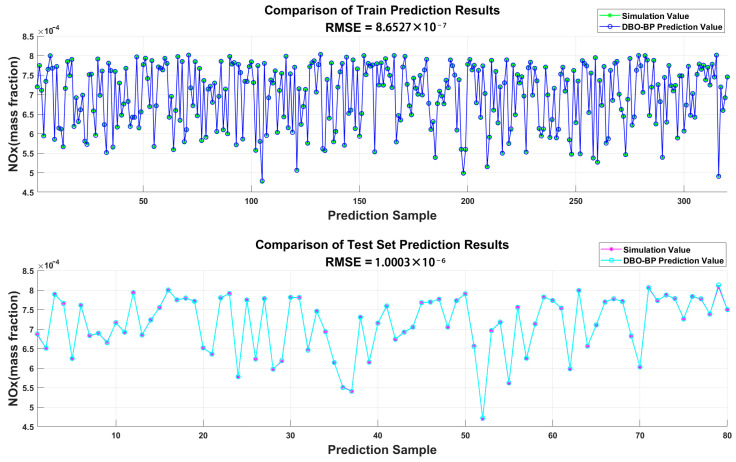
Comparison of DBO-BP prediction results for NOx.

**Figure 10:**
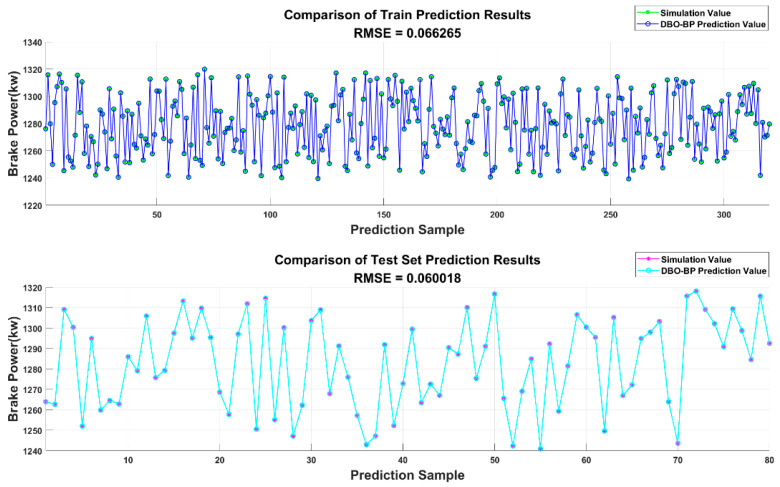
Comparison of DBO-BP prediction results for brake power.

Figure 9 and Figure 10 present a numerical comparison between the NOx and brake power outputs of the diesel engine simulation model operating at an altitude of 1000 m under standard conditions, as well as the results from the BPNN model optimized by DBO. The results indicate that the neural network predictions are in close agreement with the engine simulation data, validating the prediction accuracy and reliability of the constructed model.

## 4. Optimizing the Neural Network Prediction Model

### 4.1. Dung Beetle Optimizer

The dung beetle optimization algorithm (DBO), a nature-inspired algorithm, has recently gained popularity in the field of optimization. The algorithm simulates various behaviors of dung beetles, including rolling, dancing, foraging, stealing, and breeding, to find the optimal solution to a problem. By designing five different update rules, the DBO algorithm leverages the behavioral traits of dung beetles in different regions to achieve a deep exploration of the search space, thus avoiding the trap of local optima.

Specifically, the dung beetle’s rolling behavior is used to update positions, adjusting the search direction based on a deflection coefficient and the influence of the worst position. The dancing behavior provides new search paths when obstacles are encountered, with position updates relying on the historical information from each iteration. The egg-laying behavior adopts a boundary strategy, allowing the egg-laying position to dynamically change in response to the boundaries of the search space. The foraging behavior searches within a specific region, depending on updates from random vectors. Finally, the stealing behavior simulates the competition among dung beetles, updating their positions to explore new areas. These diverse search strategies and updated rules effectively balance global and local search capabilities, enhancing the search power during the optimization process and improving the overall performance of the algorithm.

In the DBO algorithm, each dung beetle group consists of four agents, each corresponding to one of the four distinct behaviors exhibited by the dung beetle. These behaviors form the four candidate solutions in each iteration. Through the dynamic updates of each behavior, the algorithm continuously refines the current position, ultimately converging to the optimal solution. The algorithm terminates when the predefined stopping criteria are met. The pseudocode for the DBO algorithm is presented in Algorithm 1.
**Algorithm 1:** Pseudocode for the DBO algorithm.Require: The maximum iterations T_max_, the size of the particle’s population N. Ensure: Optimal position X^b^ and its fitness value f_b_. 1: Initialize the particle’s population i←1, 2, ……, N and define its relevant parameters 2: while (t ≤ T_max_) do 3:    for i←1 to N do 4:      if i == ball-rolling dung beetle then 5:        δ = rand(1); 6:        if δ < 0.9 then 7:          Select α value by Algorithm 1; 8:          Update the ball-rolling dung beetle’s position by using (1); 9:        else 10:         Update the ball-rolling dung beetle’s position by using (2); 11:       end if 12:     end if 13:     if i == brood ball then 14:       Update the brood ball’s position; 15:     end if 16:     if i == small dung beetle then 17:       Update the small dung beetle’s position by using (6); 18:     end if 19:     if i == thief then 20:       Update the position of the thief by using (7); 21:     end if 22:   end for 23:   if the newly generated position is better than before then 24:     Update it; 25:   end if 26:   t = t + 1; 27: end while 28: return X^b^ and its fitness value f_b_

### 4.2. Neural Network Prediction Model Optimization Process

In the construction of the neural network prediction model, the BPNN is used as the foundational method. Since the engine prediction model involves various complex nonlinear factors, the BPNN, with its excellent ability to model nonlinear relationships, is well suited for effectively representing these intricate dynamics. This enables significant improvements in the accuracy of the predictions.

In this study, the DBO algorithm is applied to improve the dynamic binary optimization within the BPNN, with a focus on optimizing the network’s weights and bias parameters. The DBO algorithm enhances the efficiency of adjusting the weights and biases of the BPNN, thereby improving the overall training performance of the network.

In the entire training process of the prediction model, the initial network model of the BP neural network, along with its weights and bias parameters, is first established. The DBO algorithm represents each virtual dung beetle as a possible configuration of weights and biases. The DBO algorithm performs a global search under dynamic binary optimization conditions to attempt to find the optimal parameter combination, which is then returned to the neural network model. The algorithm updates the pheromone concentration based on the quality of the search solutions, enhancing or weakening the search directions of other beetles, thereby further improving the optimization efficiency. Finally, the optimal weights and bias parameters are passed back to the BPNN to enhance the model’s performance.

In the process of searching for weights and biases, the DBO algorithm designs four roles. The first is the rolling dung beetle, whose behavior is represented as follows:

(15) $$\begin{array}{cc} x_{i}\left(t+1\right) & =x_{i}\left(t\right)+\alpha \times k\times x_{i}\left(t-1\right)+b\times \Delta x,\\ \Delta x & =|x_{i}\left(t\right)-X^{w}| \end{array}$$

denotes the position information of the *i*th dung beetle at the *t*th iteration. Both k and b are constant values representing the deflection coefficients, and are natural coefficients assigned ±1. X^w^ represents the worst position, with Δx simulating the impact of the environment.

When the rolling dung beetle encounters an obstacle, it will acquire a new path through a dancing behavior, which is represented as follows:

(16) $$x_{i}\left(t+1\right)=x_{i}\left(t\right)+tan\left(\theta \right)|x_{i}\left(t\right)-x_{i}\left(t-1\right)|$$

The $|x_{i}\left(t\right)-x_{i}\left(t-1\right)|$ represents the positional difference during each iteration.

The reproductive behavior of the dung beetle is represented as follows:

(17) $${\mathrm{Lb}}^{*}=\max\left(X^{*}\times \;1-R,Lb\right),\;{Ub}^{*}=min\left(X^{*}\times \;1+R,Ub\right)\;B_{i}\left(t+1\right)=X^{*}+\;b_{1}\times \left(B_{i}\left(t\right)-{Lb}^{*}\right)+b_{2}\times \left(B_{i}\left(t\right)-{Ub}^{*}\right)\;\;$$

${Lb}^{*}$ and ${Ub}^{*}$ represent the lower and upper bounds for spawning. Lb and *Ub* denote the lower and upper limits of the optimization target. The position of each brood balls during each update. Each iteration is represented by $B_{i}\left(t\right)$, with random vectors *b*_1_ and *b*_2_.

The foraging behavior of the young dung beetle is represented as follows:

(18) $$x_{i}\left(t+1\right)=x_{i}\left(t\right)+C_{1}\times \left(x_{i}\left(t\right)-Lb^{b}\right)+C_{2}\times \left(x_{i}\left(t\right)-Ub^{b}\right)\;\;$$

*x_i_*(*t*) represents the position of the *i*th young dung beetle at the *t*th iteration, where *C*_1_ is a random number following a normal distribution, and *C*_2_ is a random vector within the range (0, 1).

The stealing behavior of the dung beetle is represented as follows:

(19) $$x_{i}\left(t+1\right)=X^{b}+S\times g\times \left(|x_{i}\left(t\right)-X^{*}|+|x_{i}\left(t\right)-X^{b}|\right)$$

**Figure 11:**
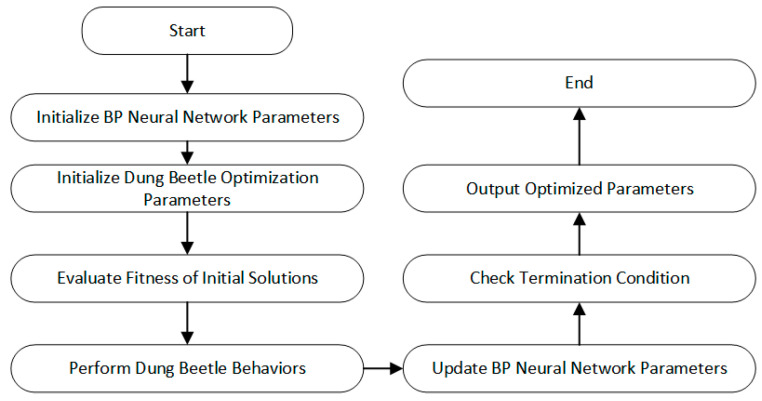
DBO-BP Flowchart.

*x_i_*(*t*) represents the position of the *i*th thief at the *t*th iteration, *g* is a random vector of size 1 × D following a normal distribution, and *S* represents a constant value. These four behaviors provide four candidate solutions for each iteration of the neural network. By continuously updating the positions, the termination criteria are met, and the best position of the dung beetle is output, which corresponds to the optimal values of the neural network’s weights and biases. Figure 11 illustrates the flowchart of the DBO algorithm optimizing the BPNN.

## 5. Multi-Objective Optimization

In this study, considering the high time cost associated with using the GT-Power simulation model for experiments, the previous section optimized the diesel engine neural network prediction model using the DBO algorithm. With the introduction of surrogate models, this section further employs multi-objective optimization methods to significantly improve experimental efficiency and reduce the reliance on computational resources in traditional experimental processes. This approach provides an efficient solution in high-time-cost environments by effectively shortening the computational time during the optimization process.

Since both input and output variables are multiples, multi-objective optimization algorithms are required for optimization. Currently, common multi-objective optimization algorithms include multi-objective particle swarm optimization (MOPSO), multi-objective ant colony optimization (MOACO), and multi-objective differential evolution (MODE), among others. Given the range of diesel engine parameters and the nonlinear nature of the outputs, compared to other multi-objective optimization algorithms, NSGA-II demonstrates a higher computational efficiency when handling multi-objective optimization problems and is capable of dealing with relatively large numbers of objectives. It is one of the most widely used multi-objective optimization algorithms today.

### 5.1. NSGA-Ⅱ (Non-Dominated Sorting Genetic Algorithm)

**Figure 12:**
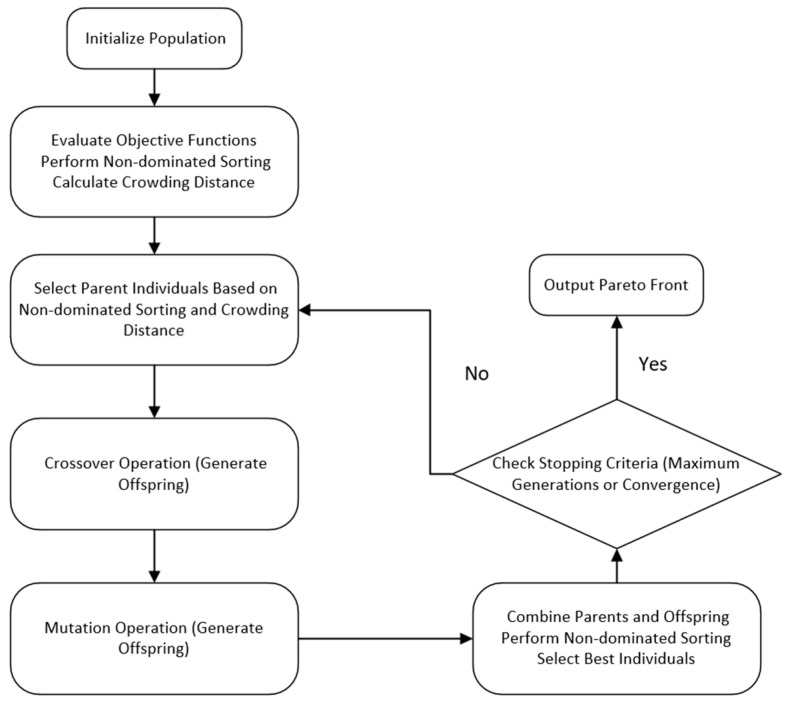
NSGA-II optimization flowchart.

After the establishment of the diesel engine surrogate model, the multi-objective optimization is performed using NSGA-II. The optimization process is shown in Figure 12. NSGA-II achieves multi-objective optimization by combining the core operations of genetic algorithms—selection, crossover, and mutation. The key feature of the algorithm lies in the non-dominated sorting, which classifies the solution set into different levels, prioritizing solutions that are not dominated by any other solution. Additionally, the crowding distance is used to maintain the diversity of the solutions, preventing the convergence to local optima.

The optimization process begins with the random generation of an initial population. Each individual is subjected to non-dominated sorting and crowding distance calculation in every generation. The parents are then selected, and a crossover and mutation are performed to generate offspring. Through continuous iterations, the algorithm progressively converges towards the optimal solution set in the objective space until the stopping criteria are met. The design of NSGA-II ensures that, during the solution process, both the solution quality is improved, and the diversity of solutions is maintained, effectively addressing the goal conflicts and trade-offs in multi-objective optimization problems.

The specific optimization steps for multi-objective optimization using NSGA-II are as follows:

(1) Establish an optimization model based on NSGA-II.
(2) Set global variables: the population size N is set to 100, the maximum number of iterations is set to 20, and the mutation probability is set to 0.8. The termination condition is either reaching the maximum number of iterations or the algorithm convergence.
(3) Randomly generate the three selected input variables (INJ, SOI, ICT) within their value ranges as the initial population P. Each individual in the initial population consists of the selected input variables.
(4) Perform non-dominated sorting: for any two individuals x_i_ and x_j_, determine if x_i_ dominates x_j_, check if, for all objectives k, f_k_(x_i_) ≤ f_k_(x_j_) and at least one objective f_k_(x_i_) < f_k_(x_j_). Sort the entire population based on the dominance relation and divide the population into multiple non-dominated layers. Then, calculate the dominance degree of each individual and assign them to each dominance layer.
(5) Compute the crowding distance: for each objective k, first, sort the solutions in the population based on their objective values, and then, calculate the crowding distance for each individual.
(6) Select parent individuals through the selection operation. Generate new offspring individuals by crossover from the selected parents. The individuals selected in the selection operation undergo a mutation to generate new individuals.
(7) Combine the parent and offspring populations. Use non-dominated sorting and crowding distance to select the top N optimal solutions and update the population.
(8) Check the termination condition (either reaching the pre-set number of iterations or little improvement in the population). If met, stop the algorithm and return the Pareto optimal solution set.

In this optimization process, the BPNN prediction model optimized by the DBO algorithm is frequently called the objective function. Every time a new individual is generated, the DBO-BPNN prediction model is called to calculate the objective values, guiding the optimization process and continuously approaching the Pareto optimal solution of the multi-objective optimization problem.

### 5.2. Analysis of Optimization Results

**Figure 13:**
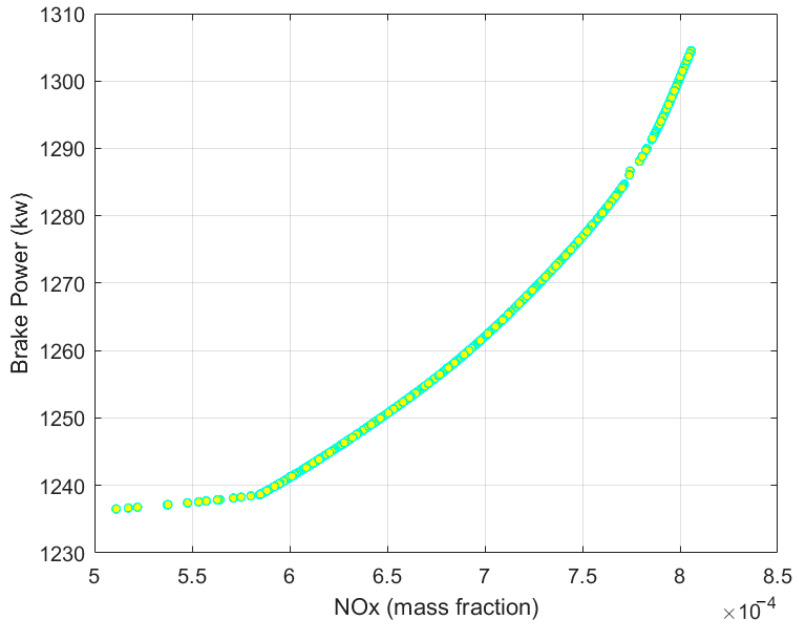
Pareto front distribution.

The Pareto front distribution of the diesel engine’s multi-objective optimization under standard conditions at an altitude of 1000 m is shown in Figure 13. This Pareto front can be used to assess the optimal performance of the diesel engine under different altitude conditions. The solutions along the Pareto front represent the best trade-offs between competing objectives, such as engine brake power and NOx emissions, allowing for a comprehensive evaluation of the engine’s performance across various operational scenarios.

After obtaining the Pareto front solutions, it is necessary to analyze the trade-off between NOx emissions and torque to select the optimal solution that best meets the target requirements. For this purpose, the Technique for Order Preference by Similarity to Ideal Solution (TOPSIS) method is used to perform a comprehensive evaluation of the Pareto front solutions. This method evaluates each solution point by calculating the distance to the ideal solution and the negative ideal solution. The solution that is closest to the ideal solution and furthest from the negative ideal solution is prioritized, and this is used to determine the optimal solution.

TOPSIS allows for selecting the most preferred solution based on its proximity to the ideal and negative ideal points, helping in making a balanced decision considering both NOx emissions and brake power performance.

### 5.3. Comparison and Analysis of the Neural Network Prediction Model and Optimal Results Under Different Altitude Conditions

In the previous section, a neural network prediction model for the diesel engine under standard conditions at an altitude of 1000 m was developed, and the corresponding optimal solution set was obtained, which largely met the expectations of the experiment. To assess the applicability of the selected algorithm under different altitude conditions, and considering the practical operating environment of the high-altitude dual-source power locomotive, experimental analyses were conducted for altitudes of 0 m, 1000 m, 22,000 m, 2800 m, 3500 m and 4000 m.

The prediction accuracy of the DBO-BP neural network model established under different altitude conditions for the two target outputs is shown in Table 4 and Table 5. It can be observed that, although the fit varies under different operating conditions, in most cases, the model maintains a high level of fit. This indicates that the constructed neural network prediction model has a strong explanatory capability for the data. It can be inferred that, under different altitude conditions, the neural network model can provide a good prediction performance, with high accuracy and robustness.

**Figure 14:**
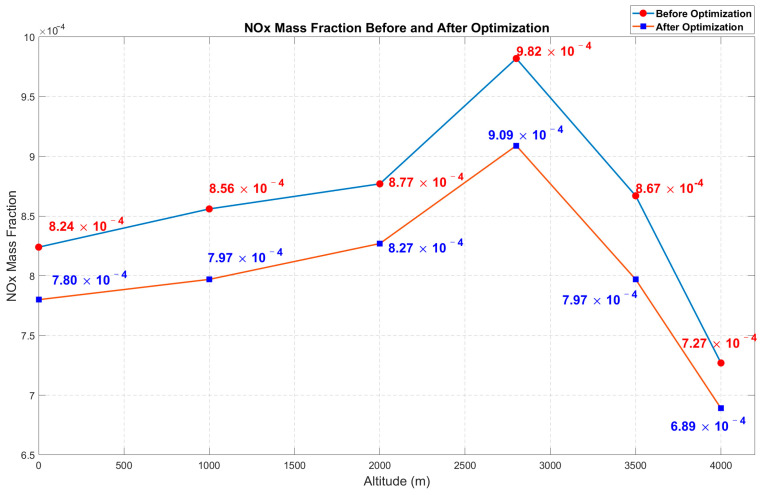
Comparison chart of NOx emissions before and after optimization.

**Figure 15:**
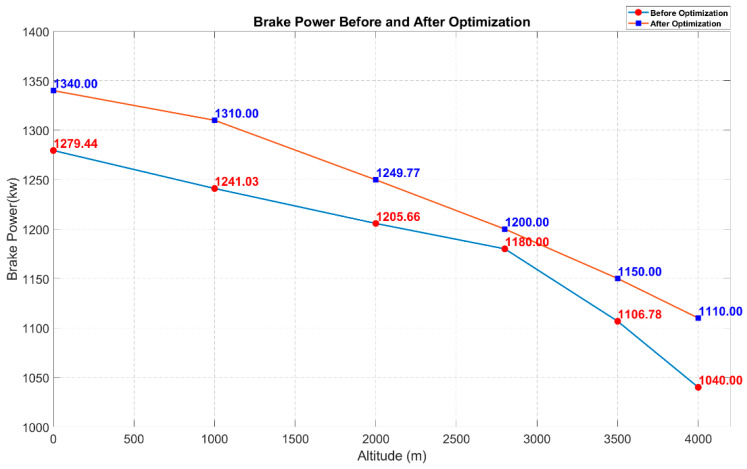
Comparison chart of brake power before and after optimization.

Under different altitude conditions, the virtual diesel engine simulation model for standard operating conditions was sampled at 400 working points using Optimal Latin Hypercube Sampling. A neural network prediction model was established and multi-objective optimization was performed using NSGA-II. The optimization results are compared in Figure 14 and Figure 15. The selected optimization results in the figures represent the optimal solutions obtained by balancing the Pareto front at different altitudes, aiming to minimize NOx emissions while maintaining a high brake power. In addition, simulation calculations were performed to determine the range of NOx mass fractions at 400 operating points across different altitudes, providing data support for the subsequent detection range of the NOx sensor in the locomotive’s diesel engine.

Table 6 shows the optimal input solutions selected after the trade-offs from the Pareto front and the verification of the corresponding output results. The optimization results shown in Figure 14 and Figure 15 indicate that the optimization algorithm selected in this paper is capable of determining the optimal behavior of the diesel engine. Improvements are observed across various altitude environments in the plateau dual-source power locomotive, suggesting that the experimental process and methods chosen in this study contribute to the optimization of the diesel engine. This highlights the potential of artificial intelligence algorithms in the optimization of diesel engines.

## 6. Conclusions

This paper conducts an optimization analysis of the internal combustion engine of a dual-source locomotive at different altitudes, as well as establishes a diesel engine dataset sample. For the first time, the DBO algorithm is combined to optimize the neural network prediction model, creating an efficient diesel engine surrogate model. Additionally, for the first time, the NSGA-II algorithm is used to perform the multi-objective optimization of the diesel engine by calling in the DBO-optimized neural network prediction model. By integrating artificial intelligence optimization algorithms, the diesel engine achieves increased output power and reduced emissions under the influence of different altitudes, and the following conclusions are drawn:

(1) Impact of Altitude on Emissions and Power: As the altitude increases due to atmospheric pressure and temperature variations, the trends in diesel engine power and nitrogen oxide (NOx) emissions differ. NOx emissions initially increase and then decrease, while power consistently decreases with rising altitudes.
(2) Engine Simulation and Coupled Modeling: In terms of engine simulation modeling and coupled simulation, the use of GT-Power combined with Matlab for co-simulation successfully constructed the diesel engine simulation model. This model provided the dataset necessary for the subsequent training of the neural network prediction model.
(3) Optimization of Neural Network Algorithms: Based on neural network training, the DBO optimization algorithm was employed to enhance the prediction accuracy significantly. This optimization method is applied to engine optimization under varying altitude conditions for the first time, demonstrating its substantial potential. The DBO algorithm exhibits strong adaptability, effectively handling multiple parameter values for the diesel engine, improving model training, and enhancing prediction accuracy.
(4) Multi-objective Optimization: The traditional NSGA-II algorithm was selected for multi-objective optimization, offering numerous advantages in diesel engine optimization. These include powerful multi-objective processing capabilities, the ability to handle complex constraints without pre-set objective weights, excellent global and local search performance, and efficient computational ability. With extensive engineering application experience, the NSGA-II algorithm achieved a good balance during the optimization process, significantly improving the diesel engine’s performance.

Through this study, we propose a new optimization neural network prediction model method and a multi-objective optimization approach aimed at improving diesel engine performance and reducing emissions in high-altitude environments. The research shows that this method determines the optimal solution by the multi-objective optimization of several key engine parameters, effectively enhancing the engine’s power output and reducing nitrogen oxide emissions in high-altitude areas. As a result, it addresses the performance degradation caused by low pressure, low oxygen, and low temperature conditions. This achievement provides both theoretical foundation and technical support for the design and operation of diesel engines in high-altitude regions.

## Figures and Tables

**Table 1:** Diesel engine parameters.

Engine Type	Four-Stroke Inline 6-Cylindere
Bore	265 mm
Stroke	300 mm
Intake Valve Opening	47° CA
Intake Valve Closing	39.3° CA
Peak Pressure Limit	180 bar
Compression Ratio	15.4

**Table 2:** Different environmental conditions at different altitudes.

Altitude (m)	Temperature (K)	Pressure (hPa)
0 m (Sea Level)	288.15	1013.25
1000 m	281.65	899.8
2000 m	275.15	794.9
2800 m	270.0	705.5
3500 m	265.4	657.6
4000 m	267.15	611.3

**Table 3:** Optimization of the number of different hidden neurons.

	5 Neuron	10 Neuron	20 Neuron	30 Neuron
Training Time	53.37 s	75.67 s	102.29	183.85
R^2^	90.99	98.99	97.59	98.89

**Table 4:** The accuracy of the neural network prediction model for NOx at different altitudes.

Altitude (m)	R^2^ of the NOx Training Set	R^2^ of the NOx Test Set	MAE of NOx Training Set	MAE of NOx Test Set
0 m (Sea Level)	0.9861	0.9812	5.78 × 10^−8^	6.66 × 10^−8^
1000 m	0.9799	0.9697	6.38 × 10^−8^	5.80 × 10^−8^
2000 m	0.9688	0.9754	9.38 × 10^−8^	7.40 × 10^−8^
2800 m	0.9696	0.9596	8.13 × 10^−6^	3.17 × 10^−6^
3500 m	0.9433	0.9855	9.65 × 10^−8^	8.83 × 10^−8^
4000 m	0.9812	0.9864	4.88 × 10^−8^	9.28 × 10^−8^

**Table 5:** The accuracy of the neural network prediction model for brake power at different altitudes.

Altitude (m)	R^2^ of the Power Training Set	R^2^ of the Power Test Set	MAE of Power Training Set	MAE of Power Test Set
0 m (Sea Level)	0.9756	0.9885	1.49	1.1634
1000 m	0.9861	0.9798	0.4731	2.2233
2000 m	0.9985	0.9645	2.3484	3.8874
2800 m	0.9189	0.9453	1.54	1.2666
3500 m	0.9413	0.9878	3.5874	4.2673
4000 m	0.9854	0.9742	7.76 × 10^−8^	6.26 × 10^−8^

**Table 6:** The optimal input solutions and corresponding output results selected after trade-offs from the Pareto front.

Altitude (m)	INJ (mg)	SOI (°)	ICT (K)	Brake Power (kw)	NOx (Mass Fraction)
0 m (Sea Level)	1759.63	−13	320.58	1340.00	7.80 × 10^−4^
1000 m	1689.02	−12.5	340.72	1310.00	7.97 × 10^−4^
2000 m	1757.85	−13.6	330.59	1249.77	8.27 × 10^−4^
2800 m	1587.88	−14.7	335.88	1200.00	9.09 × 10^−4^
3500 m	1458.40	−14.0	337.65	1150.00	7.97 × 10^−4^
4000 m	1666.55	−15.6	338.74	1110.00	6.89 × 10^−4^

## Data Availability

No new data were created or analyzed in this study. Data sharing is not applicable to this article.
